# Ocular Complications of Inflammatory Bowel Disease

**DOI:** 10.1155/2015/438402

**Published:** 2015-03-23

**Authors:** Rana Mady, Will Grover, Salim Butrus

**Affiliations:** ^1^Department of Ophthalmology, Georgetown University, Washington Hospital Center, 3800 Reservoir Road NW, Washington, DC 20007, USA; ^2^Department of Ophthalmology, Georgetown University and George Washington University, 900 23rd Street NW, Washington, DC 20037, USA

## Abstract

Though inflammatory bowel disease (IBD) has a specific predilection for the intestinal tract, it is a systemic inflammatory disorder affecting multiple organs, including the eye. Ocular complications directly related to IBD are categorized as primary and secondary. Primary complications are usually temporally associated with IBD exacerbations and tend to resolve with systemic treatment of the intestinal inflammation. These include keratopathy, episcleritis, and scleritis. Secondary complications arise from primary complications. Examples include cataract formation due to treatment with corticosteroids, scleromalacia due to scleritis, and dry eye due to hypovitaminosis A following gut resection. Some ocular manifestations of IBD can lead to significant visual morbidity and temporally associated complications can also be a herald of disease control. Furthermore, ocular manifestations of IBD can occasionally manifest before the usual intestinal manifestations, leading to an earlier diagnosis. Thus, it is important to understand the clinical presentation of possible ocular manifestations in order to initiate appropriate treatment and to help prevent significant visual morbidity.

## 1. Introduction

Inflammatory bowel disease (IBD) is a chronic inflammatory gastrointestinal disorder of unknown etiology and includes both ulcerative colitis (UC) and Crohn's disease (CD) [1]. Both entities are typically chronic disorders characterized by episodes of recurrent acute attacks. They present with symptoms of rectal bleeding, diarrhea, abdominal pain, weight loss, and low-grade fever. Similar clinical presentations can oftentimes make it difficult to differentiate between these two entities. UC affects the colon and rectum while CD can affect any part of the gastrointestinal tract, though CD tends to spare the rectum. UC is distinguished from CD by the location and extent of the lesions. Lesions in UC are continuous and involve only the superficial layers of the bowel wall, including the submucosa and mucosa. In CD, lesions are often discontinuous and tend to involve all layers of the bowel wall. Disease activity can result in significant morbidity and mortality. Initially, medical management including dietary changes, corticosteroids, and immunosuppressive treatment is employed. In medium and severe cases, biological treatment is first indicated. Surgery is reserved for complication of IBD.

The etiopathogeny of IBD is complex and not well known. Multiple factors may interfere in the etiology such as infections, genetics, and environmental factors. Epidemiological studies highlighting the differences in incidence of the disease among ethnic groups lend credence to a genetic basis for the disease [2–4]. For instance, IBD tends to occur more frequently in European countries and the United States than in Asian and Middle Eastern countries. IBD has also been noted to occur within families, further supporting the theory that genetics may play an important role in its pathogenesis [1]. Research in isolating specific genes associated with IBD is ongoing. Variations of the* Nod2* gene located on chromosome 16 have been correlated with an increased risk of developing Crohn's disease due to an altered innate immune response to gut flora [5–7].* Nod2* is probably only a small part of the picture, however, as the genetics of IBD are likely highly complex [8]. Historically, it has been suggested that IBD may be partially infectious in origin but this has yet to be supported by the isolation of consistent specific agents [9]. Perhaps the most important factor in IBD pathogenesis is immunological dysregulation, particularly increased expression of cytokines such as tumor necrosis factor-alpha and interferon gamma. The immunological etiology of IBD is supported by the positive response of IBD to immunomodulating therapy [4].

Extraintestinal manifestations of IBD most commonly involve the skin, joint, eyes, and hepatobiliary system. The incidence of ocular complications has been reported to range from 4 to 10%, occurring more often in CD than UC [2–4]. These include conjunctivitis, episcleritis, scleritis, marginal keratitis, anterior uveitis, retinitis, retinal vascular occlusive disease, optic neuritis, and orbital inflammatory disease [3]. Ocular involvement may either precede or follow the diagnosis of IBD [10]. Several factors are associated with an increased risk of ocular manifestations. Patients with colitis and ileocolitis tend to have a higher risk of ocular involvement compared to those with ileitis alone [3, 4]. Presence of other organ involvement also increases the risk [3, 4]. Particularly, in patients with CD and arthralgia, the risk of ocular involvement increases to 33% [11].

The pathophysiology of the extraintestinal manifestations of IBD is not well understood, but it is most likely mediated by the inflammatory nature of the disease. Several proposed mechanisms include circulating antigen-antibody complexes or autoantibody production against cellular antigens shared by the colon and extraintestinal organs [12]. Inflammation causing damage to the mucosal intestinal epithelium may allow proteins or microorganisms to pass through the intestinal barrier and cause a reactive lymphoid tissue response. This in turn results in antibody production or antigen-antibody complexes that circulate in the body and cause systemic inflammation. This immune response to a colonic antigen may explain why ocular manifestations may occur more commonly with colitis and ileocolitis than with small bowel involvement alone. Microbial pathogens, through molecular mimicry, may also contribute to the pathogenesis, though this is still being investigated. Genetic factors may also play a role in the ocular manifestations of IBD. Patients with extraintestinal manifestations of CD have a higher prevalence of HLA-B27 type leukocytes than the normal population [13].

Ocular complications are categorized as primary, secondary, and coincidental [14]. Primary complications are temporally associated with IBD exacerbations and tend to resolve with systemic treatment of the intestinal inflammation. These include keratopathy, episcleritis, and scleritis. Secondary complications arise from primary complications. Examples include cataract formation due to treatment with corticosteroids, scleromalacia due to scleritis, and dry eye due to hypovitaminosis A following gut resection. Coincidental complications occur commonly in the general population and cannot be correlated to IBD alone. These include conjunctivitis, recurrent corneal erosions, and corneal ulcer. This discussion will be primarily limited to the primary ocular complications of IBD involving the anterior segment. Ocular complaints in patients with IBD can oftentimes be nonspecific. As discussed below, certain ocular manifestations of IBD can lead to significant visual morbidity. Though IBD has a specific predilection for the intestinal tract, it is important to regard it as a systemic inflammatory disorder affecting multiple organs, including the eye. Thus, it is important to understand the clinical presentation of possible ocular manifestations in order to initiate appropriate treatment and to help prevent significant visual morbidity.

## 2. Keratopathy

Corneal disease (keratopathy) is a rare manifestation of IBD, but if it does occur the patient will present with eye pain, foreign body sensation, irritation, and very occasionally decreased vision. If occurring in isolation, there will be no eye redness or pupillary changes. Keratopathy associated with IBD presents as a subepithelial keratopathy and presents in two forms [15]. The first is described as epithelial or subepithelial small, grey dots found in the anterior cornea. The second has been described as deeper lamellar nebulous subepithelial infiltrates or scarring [15]. The presentation is typically bilateral and symmetric with the infiltrates located 2 to 3 mm inside the corneoscleral limbus. They tend not to cause visual morbidity because the lesions typically spare the central visual axis. Patients usually present with a known diagnosis of IBD but rarely the keratopathy may precede the diagnosis. There are no known reports of lesions that have been biopsied; thus the pathology and the underlying mechanism of formation of these infiltrates are not known. Corneal involvement may also occur secondarily to scleritis (see below). Physicians must pay particular attention to opacities in the peripheral cornea. These lesions stain with fluorescein dye, indicating corneal epithelium cell death, and can potentially become progressively thinner until they perforate. Topical steroids should be avoided in this situation as they can worsen thinning. Systemic immunosuppressive treatment for the IBD is usually sufficient for this condition [2].

## 3. Episcleritis 

Episcleritis is the most common ocular manifestation of IBD [3, 14]. The episclera is the blood-rich connective tissue between the sclera and conjunctiva. The clinical presentation includes episcleral injection, either sectoral or diffuse, that blanches with topical application of phenylephrine and tenderness to palpation. If the acute redness is sectoral—overlying just a portion of the eye—this most commonly represents episcleritis. However, scleritis can manifest in a sectoral pattern as well. In episcleritis, close examination of the eyes reveals patches of white sclera between dilated episcleral vessels. These vessels are superficial and mobile if manipulated with a cotton tip applicator. There is no loss of vision, change in pupillary response to light, corneal involvement, blurring of vision, or photosensitivity (photophobia). Mild to moderate pain and mild tenderness to palpation are typical. Episcleritis is associated with active CD and can be considered an indicator of active bowel disease [2–4].

Treatment of the underlying IBD is usually sufficient to resolve ocular symptoms. Infliximab in particular has been shown to be effective in treating episcleritis if associated with IBD [2]. However, the episcleritis can be treated independently as well. The first line therapies for mild cases are artificial tears and cool compresses for comfort as this condition is usually self-limited, even in idiopathic cases. If the episcleritis is more severe or does not resolve with systemic treatment of the IBD, topical NSAIDs like ketorolac are instituted. If the topical NSAIDs are not effective, topical steroids like prednisolone acetate may also be considered. In certain refractory cases, oral nonsteroidal anti-inflammatory drugs (NSAIDs) can be used. However, these cases should be managed in conjunction with the patient's gastroenterologist as NSAIDs can elicit an IBD flare and can worsen gastrointestinal problems [2]. Episcleritis is commonly confused with conjunctivitis, which is a common condition and may occur coincidently in a patient with IBD. Conjunctivitis may be caused by a number of different conditions including viral or bacterial infection, allergies, or chronic irritation. It typically manifests with less discomfort than episcleritis but often has more serious or purulent discharge. Additionally, conjunctivitis usually affects palpebral and bulbar conjunctiva, that is, the tissue on the eye and the inner aspect of the eyelid, where episcleritis affects only the tissues overlying the eye. There is no known association of conjunctivitis with IBD.

## 4. Scleritis 

Scleritis, a more rare complication of IBD, can result in severe visual morbidity. This presents with deep scleral injection that does not blanch with phenylephrine and a more severe, deep pain than episcleritis. Rarely, the scleritis can be associated with peripheral stromal corneal infiltrates [3]. In ambient light, the sclera may appear blue or violet. Scleritis, unlike episcleritis, is not always associated with active CD and can occur in otherwise quiescent IBD.  Table 1 summarizes the difference in presentation of iritis, episcleritis, and scleritis. Scleritis must be treated with aggressive systemic management including systemic steroids and NSAIDs or immunosuppressant agents typically in conjunction with the gastroenterologist. Repeated episodes of scleritis can result in scleromalacia perforans, extensive thinning of the sclera which can result in perforation. Thus, aggressive control of the underlying bowel disease is important to reduce the recurrence of scleritis.

Fifty percent of patients with scleritis have an underlying systemic disease and work-up is indicated unless there is a known underlying disease like IBD [14]. Common entities that cause scleritis are connective tissue diseases like rheumatoid arthritis, granulomatosis with polyangiitis (formerly Wegener granulomatosis), systemic lupus erythematosus, reactive arthritis, and of course IBD. Scleritis can also be associated with herpes zoster ophthalmicus, syphilis, status-postocular surgery, and gout. Uncommonly, scleritis can be associated with infections like tuberculosis. By contrast, episcleritis tends to be idiopathic but can be associated with herpes zoster virus, collagen vascular disorders, atopy, or other systemic diseases.

Differentiating between scleritis and episcleritis can sometimes be difficult. Generally, scleritis occurs in older patients, has a deeper, more severe pain, and can have a characteristic bluish hue. Scleral, episcleral, and conjunctival vessels are all injected. The scleral vessels do not blanch on application of topical phenylephrine. Scleritis can also involve the adjacent cornea. In contrast, episcleritis typically occurs in a younger age group and is less painful, and the normal hue of the eye is preserved under the injected vessels. The only vessels that are injected in episcleritis are those of the conjunctiva and its underlying episclera. These vessels can be moved with a cotton tip applicator whereas the scleral vessels are fixed to the globe. Episcleral vascular engorgement tends to show marked improvement with application of phenylephrine.

## 5. Anterior Uveitis 

The term uveitis describes a heterogeneous group of diseases characterized by inflammation of intraocular structures. It is typically grouped into anterior uveitis (iritis and iridocyclitis), intermediate uveitis which affects the vitreous, and posterior uveitis which affects the retina. If the inflammation occurs throughout the eye it is referred to as panuveitis. Patients typically present with red eyes that may have a characteristic concentration of conjunctival injection around the limbus called the perilimbal flush. One of the clinical hallmarks of the uveitis is sensitivity to light which is often the presenting complaint. Patients can also have blurred vision or headache.

Though IBD can manifest with a posterior or panuveitis, the typical presentation is an acute anterior uveitis, typically the nongranulomatous form [3]. Iritis, here used interchangeably with anterior uveitis, does not usually correlate with active bowel disease. It can occur during quiescent or active periods of intestinal inflammation and can precede the diagnosis of IBD. It is commonly associated with the skin findings of erythema nodosum and arthralgias. There is a well-established association between CD, sacroiliitis, and acute iritis. These patients tend to be HLA-B27 positive [4, 15]. Treatment initially entails topical or sub-Tenon's steroids and cycloplegics. In severe or refractory cases, systemic steroids or an immunosuppressant such as azathioprine is necessary.

## 6. Rare Ocular Manifestations of IBD 

Though ocular involvement of IBD tends to affect the anterior eye, there are myriad rare posterior and orbital manifestations that have been reported. These include retinitis and intermediate uveitis (uveitis involving the retina and vitreous, resp.), orbital inflammatory syndrome (nonspecific inflammation of the muscles, glands, and connective tissue that surround the eye), central and branch retinal artery occlusions, central retinal vein occlusions, optic neuritis (inflammation and swelling of cranial nerve II), and retinal vasculitis [2]. These entities, particularly the vascular occlusions, optic neuritis, and retinal vasculitis, can be devastating if not immediately recognized and treated.

## 7. Treatment 

Corticosteroids are the first-line therapy for most ocular complications of IBD that do not respond to treatment of an IBD flare or occur independently from the overall disease state. Typically, topical or sub-Tenon's steroids are employed in anterior uveitis and scleritis. If refractory to topical treatment, systemic corticosteroids should be considered in severe ocular inflammation. Systemic NSAIDs can also be considered. However, as previously mentioned, NSAIDs can cause an IBD flare and must be used in conjunction with the patient's gastroenterologist. If these measures fail, cytotoxic immunosuppressive agents such as azathioprine can be considered. These agents may be particularly effective in patients who are HLA-B27 positive.

An alternative to the immunosuppressive agents includes biologics like infliximab, a monoclonal antibody against tumor necrosis factor alpha, and adalimumab. It has been approved by the US Food and Drug Administration for the treatment of CD and has been shown to be effective in refractory cases of episcleritis, scleritis, and uveitis [2, 16]. Biologics for the treatment of uveitis have been increasingly studied over the past decade for IBD, seronegative spondyloarthropathies, and juvenile idiopathic arthritis with good results. Though studied, etanercept has not yet been shown to be efficacious with these conditions. Most reports of biological therapies in uveitis have been uncontrolled trials or retrospective studies and have investigated cases of uveitis refractory to immunosuppression. Due to lack of evidence thus far, the biologics are reserved for such refractory cases or if occurring in patients with other systemic symptoms that would normally be treated with biologics [17].

Specifically for IBD, infliximab is currently the only biological agent approved; however, many agents are being investigated and show promise. In normal intestinal mucosa, inflammation is regulated by a balance of proinflammatory cytokines, TNF alpha, interferon gamma, IL-1, IL-6, and IL-12, and anti-inflammatory cytokines, IL-4, IL-10, and IL-11. Therefore, each of these cytokines is a potential target for therapy. Current investigations are ongoing for targeting TNF, leukocyte adhesion, T-helper cell polarization, T-cell activation, and nuclear factor. Anti-TNF biological agents under review for Crohn's disease include CDP 571, certolizumab pegol, etanercept, onercept, and adalimumab [18]. Though most studies focus on control of intestinal inflammation, there are a few studies that show good results with infliximab for ocular involvement [19]. Further study is likely to elucidate similar improvements in ocular and other nonintestinal manifestations with other agents.

Several of the treatments of IBD and other systemic inflammatory diseases secondarily cause ocular pathology. The most common offender is steroid treatment. Systemic and topical corticosteroids lead to cataracts, specifically posterior subcapsular cataracts. Cataracts lead to progressive loss of visions that is usually correctable with cataract surgery. However, cataracts in children under the age of seven can result in irreversible vision loss. Additionally, children rarely complain of vision loss even in severe cases and, therefore, need particularly close follow-up with an ophthalmologist if being treated with steroids. Steroids also cause glaucoma in a significant portion of patients which leads to irreversible vision loss [2]. Glaucoma often has no symptoms until its final stages and is a common cause of blindness. Therefore, patients of all ages need regular glaucoma screenings if taking systemic steroids. Anticholinergic agents used for abdominal cramping associated with IBD can cause pupillary and accommodation (near vision reflex) disturbances which can be annoying to patients but cause no permanent harm. These agents rarely cause angle closure glaucoma in susceptible individuals. Cyclosporine, used in Crohn's disease, has been rarely reported to cause optic neuropathy, ophthalmoplegia, and nystagmus. Methotrexate builds up in the tears and can cause conjunctival inflammation [2].

## 8. Conclusion 

IBD, though mostly targeting the intestinal tract, is a chronic systemic disease. The underlying pathophysiology leading to the ocular manifestations of IBD is still not well understood. Ophthalmic involvement, such as uveitis, is particularly important because it can rarely precede other symptoms of the disease. Thus, it is important to ask patients presenting with an acute anterior uveitis about fever, abdominal pain, bloody diarrhea, anemia, and weight loss. If the diagnosis of IBD can be made before GI involvement becomes severe, many long term consequences of the disease can be avoided or delayed. Ocular involvement does not always coincide with active intestinal flare. However, in instances when it does, as is typical of episcleritis, this can be used as an indicator of disease activity indicating the need for more aggressive management [14]. Treatment of ocular manifestations ranges from controlling the underlying intestinal inflammation, topical steroids, systemic NSAIDS, systemic steroids, and immunosuppressant and biological agents. In refractory or severe cases, treatment with monoclonal antibodies has shown promise. In severe cases associated with active bowel inflammation, colonic surgical resection may be required to calm the ocular inflammation. An interdisciplinary approach involving the ophthalmologist, internist, and gastroenterologist best serves these patients.

## Figures and Tables

**Table 1:** Iritis versus episcleritis versus scleritis.

	Iritis	Episcleritis	Scleritis

Presentation	Perilimbal flush, photosensitivity, blurry vision	Red eye, minimal pain, blanches with phenylephrine	Red eye, deep pain, violet hue, does not blanch with phenylephrine

Treatment	Topical steroids	Observation, topical NSAIDs	Topical steroids, topical NSAIDs, systemic therapy

Differential diagnosis for underlying disease	Idiopathic, trauma, HLA-B27 associated systemic diseases like IBD, other systemic conditions, postoperative	Idiopathic, herpes zoster, rarely systemic disease	Connective tissue disease, herpes zoster, syphilis, postoperative, gout
